# Altered Synaptic Vesicle Release and Ca^2+^ Influx at Single Presynaptic Terminals of Cortical Neurons in a Knock-in Mouse Model of Huntington’s Disease

**DOI:** 10.3389/fnmol.2018.00478

**Published:** 2018-12-24

**Authors:** Sidong Chen, Chenglong Yu, Li Rong, Chun Hei Li, Xianan Qin, Hoon Ryu, Hyokeun Park

**Affiliations:** ^1^Division of Life Science, The Hong Kong University of Science and Technology, Kowloon, Hong Kong; ^2^Department of Physics, The Hong Kong University of Science and Technology, Kowloon, Hong Kong; ^3^Department of Neurology, Boston University School of Medicine, Boston, MA, United States; ^4^State Key Laboratory of Molecular Neuroscience, The Hong Kong University of Science and Technology, Kowloon, Hong Kong

**Keywords:** Huntington’s disease, synaptic vesicle release, calcium influx, real-time imaging, presynaptic terminal

## Abstract

Huntington’s disease (HD) is an inherited neurodegenerative disorder caused by the abnormal expansion of CAG repeats in the *huntingtin* (*HTT*) gene, which leads to progressive loss of neurons starting in the striatum and cortex. One possible mechanism for this selective loss of neurons in the early stage of HD is altered neurotransmission at synapses. Despite the recent finding that presynaptic terminals play an important role in HD, neurotransmitter release at synapses in HD remains poorly understood. Here, we measured synaptic vesicle release in real time at single presynaptic terminals during electrical field stimulation. We found the increase in synaptic vesicle release at presynaptic terminals in primary cortical neurons in a knock-in mouse model of HD (zQ175). We also found the increase in Ca^2+^ influx at presynaptic terminals in HD neurons during the electrical stimulation. Consistent with increased Ca^2+^-dependent neurotransmission in HD neurons, the increase in vesicle release and Ca^2+^ influx was rescued with Ca^2+^ chelators or by blocking N-type voltage-gated Ca^2+^ channels, suggesting N-type voltage-gated Ca^2+^ channels play an important role in HD. Taken together, our results suggest that the increased synaptic vesicles release due to increased Ca^2+^ influx at presynaptic terminals in cortical neurons contributes to the selective neurodegeneration of these neurons in early HD and provide a possible therapeutic target.

## Introduction

Huntington’s disease (HD) is an autosomal dominant neurodegenerative disorder caused by an increase in CAG trinucleotide repeats in the *huntingtin* (*HTT*) gene, giving rise to an expanded polyglutamine (polyQ) domain in the N-terminal of the encoded HTT protein (MacDonald et al., 1993). The main clinical symptoms of HD include severe involuntary motor dysfunction, psychiatric disturbance, cognitive impairment, and eventual death (Dayalu and Albin, 2015). Although the *HTT* gene is ubiquitously expressed throughout the human body, the medium spiny neurons (MSNs) in the striatum and the pyramidal neurons in the cortex are most vulnerable neurons in HD (Vonsattel and DiFiglia, 1998).

It has been suggested that the selective loss of these neurons in HD may be associated with altered synaptic transmission (Klapstein et al., 2001; Cepeda et al., 2003; Li et al., 2003; Smith et al., 2005; Raymond et al., 2011; Tyebji and Hannan, 2017; Virlogeux et al., 2018), reduced support of brain-derived neurotrophic factor (BDNF; Zuccato and Cattaneo, 2009, 2014; Park, 2018; Yu et al., 2018), mitochondrial dysfunction (Carmo et al., 2018; Franco-Iborra et al., 2018), and altered Ca^2+^ regulation (Bezprozvanny, 2009; Miller and Bezprozvanny, 2010; Raymond, 2017). In particular, altered synaptic transmission has been investigated as a pathogenic mechanism and as a possible therapeutic target in HD (Raymond, 2017; Tyebji and Hannan, 2017). Some studies have focused on postsynaptic terminals, which are generally related to neuronal death in the striatum (Raymond, 2017). For example, increased activity of extrasynaptic N-methyl-D-aspartate receptors (NMDARs) was suggested to lead to the degeneration of striatal neurons (Milnerwood et al., 2010; Botelho et al., 2014; Plotkin et al., 2014). An increased intracellular Ca^2+^ concentration due to increased NMDAR activity and/or other Ca^2+^ sources, including the InsP_3_R1 (inositol 1,4,5-trisphosphate receptor type 1) and store-operated Ca^2+^ entry, was also suggested to underlie the striatal neurodegeneration in HD (Tang et al., 2003, 2004; Wu et al., 2016). Activation of the NMDAR can be induced by the release of neurotransmitters from presynaptic terminals (Blanton et al., 1990; Atasoy et al., 2008). In this respect, it is interesting to note that mutant HTT protein has been reported to alter the exocytosis of presynaptic vesicles (DiFiglia et al., 1995; Morton et al., 2001; Romero et al., 2008; Joshi et al., 2009). Recently, reconstituted corticostriatal synapses formed in microfluidics devices showed that presynaptic terminals from cortical neurons contribute to HD (Virlogeux et al., 2018). However, the release of neurotransmitters at single presynaptic terminals as an input of corticostriatal synapses in HD remains poorly understood.

Using real-time imaging of single presynaptic terminals containing FM 1–43-labeled synaptic vesicles, we measured synaptic vesicle release in real time at single presynaptic terminals during electrical field stimulation. We found the increase in synaptic vesicle release at presynaptic terminals in primary cortical neurons in a knock-in mouse model of HD (zQ175). We also found the increase in Ca^2+^ influx at presynaptic terminals in HD neurons during the electrical stimulation. The increased release and Ca^2+^ influx in cortical neurons of heterozygous zQ175 were reduced by loading the neurons with BAPTA-AM (a Ca^2+^ chelator) or treating neurons with ω-conotoxin GVIA (a selective N-type voltage-gated Ca^2+^ channel blocker).

## Materials and Methods

### Mice

The HD knock-in mice (zQ175) were obtained from Jackson Laboratories and were maintained in the Animal and Plant Care Facility at the Hong Kong University of Science and Technology (HKUST). Heterozygous mice were used for breeding. All procedures were approved by the Department of Health, Government of Hong Kong.

### Primary Cultures of Cortical Neurons

HD and wild-type (WT) cortical neurons were obtained from primarily neocortical tissue of postnatal day 0 (P0) heterozygous pups and littermates. Approximately 10^5^ neurons were cultured on each glass coverslip in 24-well plates as described previously (Liu and Tsien, 1995). Three days after plating the neurons (days in vitro 3, DIV3), 20 μM 5-Fluoro-2’-deoxyuridine (FUDR, Sigma) was added to inhibit the proliferation of glia (Leow-Dyke et al., 2012). The neurons were incubated at 37°C in humidified air containing 5% CO_2_ for at least 14 days, and imaging and patch-clamp experiments were performed between DIV14 and DIV21.

### Immunofluorescence

Cultured cortical neurons were fixed in 4% paraformaldehyde in phosphate-buffered saline (PBS) at room temperature for 10 min. After washing three times with ice-cold 0.1 M PBS, the neurons were permeabilized with PBS containing 0.1% Triton X-100 at room temperature for 10 min. After washing and blocking, 50 μl of primary antibody mixtures containing polyclonal anti-microtubule-associated protein 2 [anti-MAP2; 1:500; Ab5392 (Abcam)] together with the monoclonal anti-vesicular glutamate transporter 1 [anti-VGLUT1; 1:500; MAB5502 (Millipore)], monoclonal anti-glutamic acid decarboxylase 67 [anti-GAD67; 1:500; MAB5406 (Millipore)], monoclonal anti-GFAP [1:500; MAB360 (Millipore)], monoclonal anti-polyQ [1:500; MAB1574 (Millipore)], or monoclonal anti-ubiquitin [1:200; ab7780 (Abcam)] were added to each coverslip, and the neurons were incubated in a cold room overnight. The following day, Alexa 546-conjugated (A11040, Abcam) and Alexa 647-conjugated secondary antibodies (A31571, Abcam) were added. After washing with PBS three times, 50 μl DAPI (300 nM) was added to each coverslip, and the coverslips were mounted on glass slides. Fluorescence images were obtained using a Zeiss LSM 710 microscope. The percentage of VGLUT1 positive neuron was calculated by 100 × [(the number of VGLUT1-positive cells)/(the number of MAP2-positive cells)]. The percentage of GAD67-positive neurons was calculated by 100 × [(the number of GAD67-positive cells)/(the number of MAP2-positive cells)]. The percentage of GFAP-positive cells was calculated by 100 × [(the number of GFAP-positive cells)/(the number of MAP2-positive cells + the number of GFAP-positive cells)]. The number of VGLUT1-positive, GAD67-positive, and GFAP-positive cells was calculated in a blinded fashion.

### Imaging of FM 1–43-Labeled Synaptic Vesicles

FM 1–43 was loaded into synaptic vesicles in cultured cortical neurons by applying 1-ms field stimuli at 10 Hz for 120 s in the presence of 16 μM FM 1–43 (T35356, Thermo Fisher Scientific) at 37°C using a parallel platinum electrode connected to an SD9 Grass Stimulator (Grass Technologies). The chamber was perfused for 10 min with ACSF solution containing (in mM): 120 NaCl, 4 KCl, 2 CaCl_2_, 2 MgCl_2_, 10 D-Glucose, and 10 HEPES (300–310 mOsm, pH 7.2–7.4 with NaOH). The experiments were performed similarly as described previously (Zhang et al., 2007; Park et al., 2012). Time-lapse images were acquired for 200 s at 1 Hz with an exposure time of 0.1 s using an iXon Ultra EMCCD camera (Andor camera). The experimental setup was made up of several pieces of equipment as described previously (Alsina et al., 2017). The stimulator, beam shutter, and EMCCD camera were synchronized with a trigger from the camera *via* a Digidata 1550 (Molecular Devices). Clampex (Molecular Devices) was used to generate the stimulation protocol. An inverted IX-73 microscope (Olympus) was used with a 100× UPlanSApo oil-immersion objective (Olympus). A 532-nm laser (CrystaLaser) was used to illuminate FM 1–43 with a dichroic mirror (ZT532rdc) and emission filter (ET595/50m). The normalized fluorescence of FM 1–43 was calculated as the fluorescence intensity in a region of interest (ROI) relative to the mean of resting fluorescence intensity in that ROI during the first 20 s (*F/F*_0_). The fluorescence intensity in a ROI was analyzed using MetaMorph software (Molecular Devices) and the normal fluorescence was calculated using customer-made MATLAB (MathWorks, Inc.) program. Average traces of normalized fluorescence were calculated using the average signal from all analyzed individual boutons. Fluorescence loss was calculated by subtracting the average normalized intensity in the final 60 s after stimulation and is expressed as a percentage. The time constant of FM 1–43 destaining was calculated by fitting the data from 20 s through 140 s to an exponential decay using a custom-written MATLAB program. The fitting with *R* > 0.8 was included for comparison of time constants of FM 1–43 destaining between WT and HD cortical neurons.

### Electrophysiology

The paired-pulse ratio (PPR) between two evoked excitatory postsynaptic currents (eEPSCs) was measured in cultured cortical neurons after DIV14. Neurons were voltage clamped in the whole-cell configuration and recorded using a MultiClamp 700B amplifier (Molecular Devices) and a Digidata 1440a digitizer (Molecular Devices). Recordings were performed at room temperature under continuous perfusion of ACSF solution. Glass pipettes with a resistance of 3–5 MΩ were filled with an intracellular solution containing (in mM): 140 K-gluconate, 2 MgCl_2_, 0.5 EGTA, 10 HEPES, and 0.5 Mg-ATP (300–310 mOsm, pH 7.3 with NaOH). The recorded neuron was held at −70 mV, and the stimulating electrode was positioned 100–200 μm from the recorded neuron. Two consecutive stimuli were applied at increasing intervals of 50, 100, 150, and 200 ms. PPR was calculated as the ratio between the second eEPSC amplitude and the first eEPSC amplitude.

### Ca^2+^ Imaging

We performed Ca^2+^ imaging at single presynaptic terminals in cultured neurons using Cal-520-AM (21130, AAT Bioquest). A stock solution of 1 mM Cal-520-AM was prepared in dimethyl sulfoxide (DMSO) and stored at −20°C. The stock solution of Cal-520-AM was added to the culture medium at the final concentration of 1 μM, and neurons were incubated for 30 min. During the incubation, neurons were also incubated with FM 4–64 (T13320, Thermo Fisher Scientific) at a final concentration of 10 μM to label presynaptic terminals (Gaffield and Betz, 2006). Loaded neurons were then transferred to the imaging chamber and perfused continuously with ACSF for more than 20 min before imaging. FM 4–64 fluorescence was imaged using the frame-transfer mode for 10 frames with a 532-nm laser (CrystaLaser) with a dichroic mirror (ZT532rdc) and emission filter (ET665lp). Cal-520 fluorescence was acquired using a 488-nm laser (CrystaLaser) with a dichroic mirror (ZT488rdc) and emission filter (ET525/50m). Time-lapse imaging of Cal-520 was acquired similar to FM 1–43 imaging. Continuous imaging of Cal-520 with high temporal resolution was performed using a frame-transfer mode of 10 Hz (exposure time of 0.1 s) during 0.1 Hz external stimulation. The concentration of Ca^2+^ was estimated by the relative changes in fluorescence intensity relative to baseline fluorescence of Cal-520 (Δ*F/F*_0_).

### FM 1–43 Destaining in the Presence of BAPTA-AM

FM 1–43 was loaded into synaptic vesicles as described above; 2 min after loading, the chamber was perfused with ACSF at 37°C containing 200 nM BAPTA-AM (B1205, Thermo Fisher Scientific) for 10 min. FM 1–43 imaging was then performed as described above. Data was analyzed as described above.

### FM 1–43 Destaining in the Presence of ω-Conotoxin GVIA or ω-Agatoxin IVA

FM 1–43 was loaded into synaptic vesicles as described above; 2 min after loading, the chamber was perfused with ACSF for 10 min. The perfusion solution was then switched to ACSF containing 100 nM ω-conotoxin GVIA (C-300, Alomone Labs) or 200 nM ω-agatoxin GVIA (STA-500, Alomone Labs); 2 min later, FM 1–43 imaging was performed as described above. Data was analyzed as described above.

### Ca^2+^ Imaging in the Presence of ω-Conotoxin GVIA

Neurons were loaded with Cal-520-AM and FM 4–64 as described above. After perfusion with ACSF for more than 20 min, the perfusion solution was then switched to ACSF containing 100 nM ω-conotoxin GVIA; 2 min later, FM 4–64 and Cal-520 imaging was performed as described above. Data was analyzed as described above.

### Ca^2+^ Imaging in the Presence of Calciseptine, AP5, and NBQX

Neurons were loaded with Cal-520-AM and FM 4–64 as described above. After perfusion for >20 min with ACSF containing 1 μM calciseptine, 50 μM AP5, and 10 μM NBQX, FM 4–64 and Cal-520 imaging was performed as described above. Data were analyzed as described above.

### Analysis

MetaMorph (Molecular Devices) was used to calculate fluorescence of FM 1–43 and Cal-520 within the ROIs. All data are presented as the mean ± SEM. Differences between HD and WT neurons were determined using the independent two-tailed Student’s *t*-test or the Kolmogorov–Smirnov (K-S) test (SPSS, IBM Corp.). The effect of the toxins of voltage-gated Ca^2+^ channels on the release of FM 1–43 on genotypes was analyzed using two-way ANOVA analysis (SPSS, IBM Corp.). Differences with *p* < 0.05 were considered significant.

## Results

### Excitatory Neurons Are Predominant in Primary Cortical Neurons

The loss of neurons in HD has been suggested to be associated with altered synaptic transmission (Raymond, 2017; Tyebji and Hannan, 2017). However, the release of neurotransmitters at single presynaptic terminals in HD has not been investigated in detail. To examine whether synaptic transmission is altered at single presynaptic terminals in HD neurons, we measured the release of synaptic vesicles at single presynaptic terminals in dissociated cortical neurons cultured from zQ175 mice, a recently developed knock-in mouse model of HD that is more relevant to human HD than other models in terms of genetic context and recapitulating the late onset, slow natural progression, and neuropathology of HD patients. Previous studies showed that the volume of the striatum and cortex decreases beginning at 4 months of age in heterozygous mice, and behavioral deficits appear at 4.5 months (Heikkinen et al., 2012; Menalled et al., 2012).

**Figure 1:**
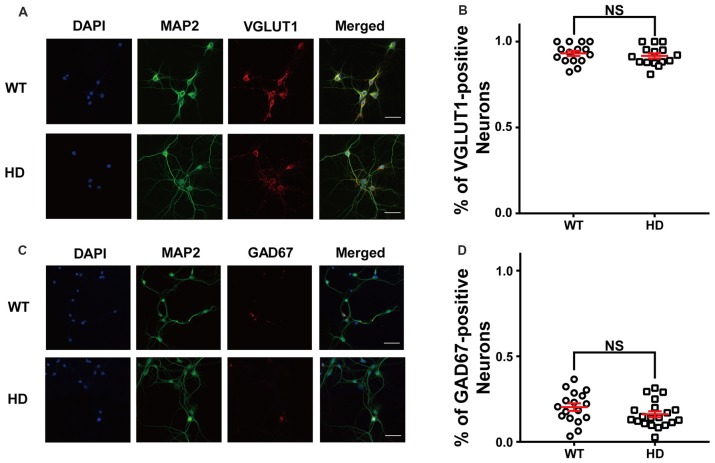
The majority of cultured cortical neurons are excitatory.** (A)** Representative confocal images of wild-type (WT) and Huntington’s disease (HD) cultured cortical neurons isolated from WT and heterozygous zQ175 mice immunostained for microtubule-associated protein 2 (MAP2; green) and vesicular glutamate transporter 1 (VGLUT1; an excitatory neuronal marker; red); the nuclei were counterstained with DAPI (blue). The scale bar represents 40 μm. **(B)** The percent of VGLUT1-positive neurons in WT and HD cortical neurons. The percentage of VGLUT1-positive neurons were higher than 90% regardless of genotype, indicating that the vast majority of neurons are excitatory. The percentage between WT and HD showed no significant differences (*p* = 0.0225, from independent two-tailed Student’s *t-test*). **(C)** Representative confocal images of cultured neurons WT and HD cortical neurons immunostained for MAP2 (green) and glutamic acid decarboxylase 67 (GAD67; an inhibitory neuronal marker; red); the nuclei were counterstained with DAPI (blue). Scale bar represents 40 μm. **(D)** The percent of GAD67-positive neurons in WT and HD neurons. The percentage of GAD67-positive neurons between WT and HD showed no significant differences (*p* = 0.136; independent two-tailed Student’s *t*-test). In this and subsequent figures, data are presented as the mean ± SEM. NS, not significant.

First, we measured the relative proportion of excitatory neurons in dissociated cortical neurons by plating low-density WT and HD cortical neurons isolated from P0 littermates; the neurons were then cultured until mature, functional synapses were formed. After DIV14, the cultured neurons were fixed and immunostained for MAP2 (a neuron-specific marker) and VGLUT1 (a marker for excitatory synapses). Figure 1A shows representative confocal images of WT and HD cortical neurons co-stained for MAP2 and VGLUT1. Our analysis between MAP2 and VGLUT1 immunoreactivity (Figure 1B) revealed that the vast majority of primary cultured cortical neurons are excitatory (i.e., VGLUT1-positive), with no significant difference between WT and HD neurons [93 ± 1.5% (*N* = 14) vs. 92 ± 1.5% (*N* = 15), *p* = 0.225 from independent two-tailed Student’s *t*-test]. We also co-stained neurons for MAP2 and GAD67 (a marker for inhibitory neurons) in order to measure the proportion of inhibitory neurons in our cultures (Figure 1C). Our analysis confirms that only a minority of neurons are inhibitory, with no significant difference between WT and HD neurons [20 ± 2.2% (*N* = 17) vs. 16 ± 1.7% (*N* = 20), *p* = 0.136 from independent two-tailed Student’s *t*-test; Figure 1D]. We also measured the number of glial cells in our primary cultures and found that glial cells represent approximately 12% of the cells in both WT and HD neurons (12 ± 1.6% vs. 11 ± 1.5%, respectively; *N* = 10; *p* = 0.774, independent two-tailed Student’s *t*-test; Supplementary Figure S1). We also looked for the presence of inclusion bodies (a pathological hallmark of HD) in our culture system using antibodies against polyQ and ubiquitin; however, no such aggregates were observed (Supplementary Figure S2). Taken together, our immunostaining results indicate that the majority of cultured mouse cortical neurons are excitatory, consistent with previous reports (Gabbott and Somogyi, 1986; Hendry et al., 1987).

### Synaptic Vesicle Release Is Increased in HD Cortical Neurons

Next, we examined whether the mutant HTT protein affects the release of synaptic vesicles at presynaptic terminals of cortical neurons. We loaded presynaptic vesicles with FM 1–43 (a membrane-impermeable lipophilic styryl dye) using electrical field stimulation. We then measured the decrease in FM 1–43 fluorescence at single presynaptic terminals during electrical field stimulation (Betz and Bewick, 1992; Ryan et al., 1993; Gaffield and Betz, 2006; Yu et al., 2016). The loading protocol consisted of a train of 1,200 1-ms stimuli delivered at 10 Hz for 120 s, which labels the total recycling pool of synaptic vesicles (Zhang et al., 2007, 2009; Park et al., 2012), followed by extensive washing with ACSF to remove extracellular and plasma membrane-bound FM 1–43, thereby enabling us to specifically monitor FM 1–43-loaded vesicles.

**Figure 2:**
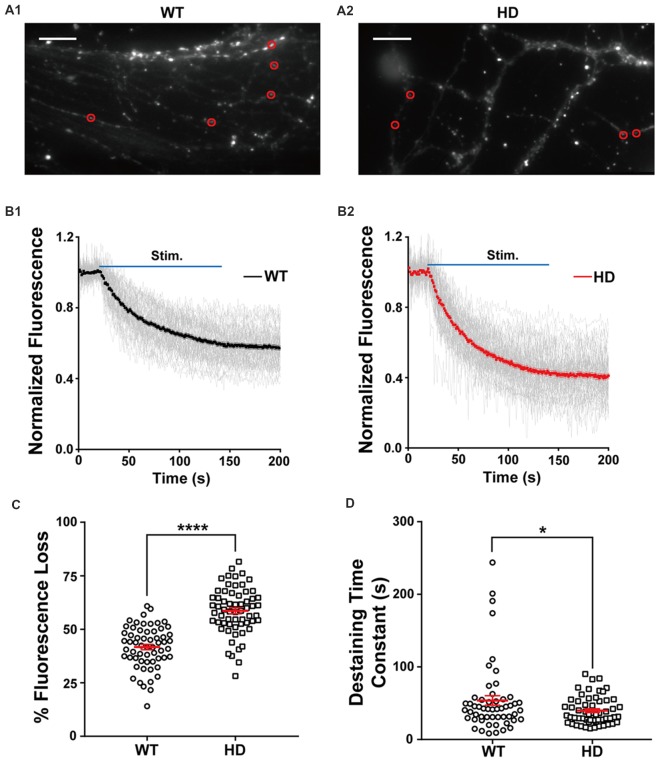
HD cortical neurons have increased synaptic vesicle release. **(A1,A2)** Representative images of WT and HD cortical neurons loaded with FM 1–43. The red circles indicate the regions of interest (ROIs) representing presynaptic terminals that were analyzed for FM 1–43 release. The scale bars represent 10 μm. **(B1,B2)** Traces of normalized fluorescence intensity showing FM 1–43 destaining during 1,200 1-ms field stimuli applied at 10 Hz for 120 s in WT (*n* = 60 boutons, *N* = 7 experiments) and HD cortical neuron (*n* = 62 boutons, *N* = 8 experiments). **(C)** The percent fluorescence loss of FM 1–43 staining in WT and HD cortical neurons. **(D)** The time constant of FM 1–43 destaining in WT and HD cortical neurons. *p < 0.05 and ****p < 0.0001 (independent two-tailed Student’s *t*-test).

Figure 2A shows typical fluorescence images of cultured WT (Figure 2A1) and HD (Figure 2A2) neurons after extensive washing, showing FM 1–43-loaded presynaptic vesicles (Ryan et al., 1993; Henkel et al., 1996). Fluorescence intensity of the FM 1–43 signal was calculated at fixed ROI (Harata et al., 2001; Jordan et al., 2005), each of which encompasses a single isolated FM 1–43-bright spot called a bouton, likely representing a single presynaptic terminal. To exclude synapses that were labeled by spontaneous neuronal activity and nonspecific labeling with FM 1–43, isolated boutons that showed destaining upon electrical field stimulation were used to measure synaptic vesicle release.

The density of functional presynaptic terminals was calculated by counting boutons labeled with FM 1–43 at the presynaptic terminal. The average bouton density was similar between cultured WT and HD cortical neurons (0.59 ± 0.008 boutons · μm^−1^ and 0.59 ± 0.014 boutons · μm^−1^, respectively; *N* = 5 experiments/genotype; *p* = 0.803), suggesting that the presence of the mutant HTT protein does not alter the number of functional presynaptic terminals in cortical neurons.

Next, we used electrical field stimulation to destain FM 1–43-loaded synaptic vesicles and measured vesicle release using real-time imaging of single presynaptic terminals. The release of vesicular FM 1–43 was reflected as a decrease in FM 1–43 intensity in each ROI. Figure 2B displays a time course of normalized fluorescence intensity in WT (Figure 2B1) and HD (Figure 2B2) cortical neurons at individual boutons during electrical field stimulation with 1,200 electrical pulses, which trigger the release of synaptic vesicles from the total recycling pool at the level of single boutons (Zhang et al., 2007, 2009; Welzel et al., 2011; Park et al., 2012; Yu et al., 2016). The normalized fluorescence intensity was calculated as the ratio of the intensity in the ROI with respect to the average intensity before stimulation (i.e., baseline fluorescence). As expected, our stimulation protocol induced the rapid release of FM 1–43 in individual presynaptic terminals in both WT and HD neurons. The degree of fluorescence loss at individual presynaptic terminals in cortical neurons was highly variable; consistent with previous reports (Murthy et al., 1997; Branco and Staras, 2009; Daniel et al., 2009), the coefficient of variation (CV) was 23% and 19% in WT and HD neurons, respectively. Nevertheless, the total loss of fluorescence was significantly higher in HD neurons compared to WT neurons [59 ± 1.3% (*n* = 62 boutons, *N* = 8 experiments for HD) vs. 42 ± 1.4% (*n* = 60 boutons, *N* = 7 for WT), *p* = 4.27E-15 from independent two-tailed Student’s *t*-test; Figure 2C] during the electrical stimulation to trigger the release of synaptic vesicles from the total recycling pool. Given that relative release probability (*P*_r_) is proportional to the relative release of lipophilic dyes (Murthy et al., 1997; Branco et al., 2008; Branco and Staras, 2009; Daniel et al., 2009), our finding of increased fluorescence loss at presynaptic terminals of HD neurons likely indicates that in HD, cortical neurons have a higher release probability compared to WT neurons.

In addition to measuring total fluorescence loss, we also measured the kinetics of vesicle release by fitting the time course of fluorescence loss with an exponential time constant (Richards et al., 2005; Daniel et al., 2009). As shown in Figure 2D, the time constant of FM 1–43 destaining was significantly smaller in HD neurons compared to WT neurons [39.9 ± 2.5 s (*n* = 57 boutons) for HD vs. 53.8 ± 6.5 s (*n* = 54 boutons) for WT, *p* = 0.0448 from independent two-tailed Student’s *t-test*], suggesting faster vesicle release at presynaptic terminals of HD cortical neurons. Taken together, these results indicate that cortical neurons expressing the mutant HTT protein have increased neurotransmission compared to WT neurons under similar stimulation conditions.

Next, we measured whether inhibitory neurotransmission is altered in inhibitory synapses by measuring the release of VGAT-cypHer5e, a fluorescent marker that selectively labels inhibitory synaptic vesicles (Hua et al., 2011). In contrast with our FM 1–43 results, WT and HD neurons were similar with respect to VGAT-cypHer5e fluorescence loss (Supplementary Figure S3), suggesting that inhibitory neurotransmission is not altered in cortical neurons expressing mutant HTT proteins. Given the relatively small number of inhibitory neurons in our cortical cultures and no alteration in the inhibitory neurotransmission in HD cortical neurons, we conclude that the mutant HTT protein primarily affects excitatory neurotransmission in cortical neurons.

### Synapses in HD Cortical Neurons Have Paired-Pulsed Depression

**Figure 3:**
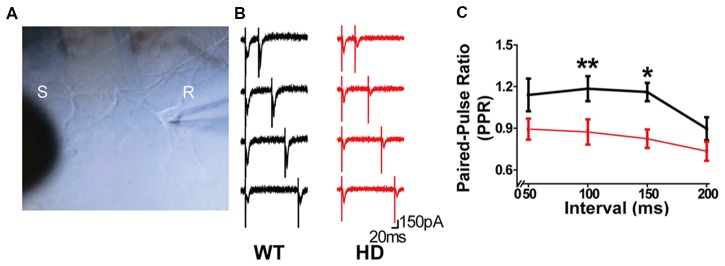
Synapses in HD cortical neurons exhibit paired-pulse depression. **(A)** Example image of cultured cortical neurons, showing the stimulating electrode (S) and recording electrode (R). **(B)** Representative traces of evoked excitatory postsynaptic currents (eEPSPs) recorded in WT and HD cortical neurons. From top to bottom, the interval between the two stimuli was 50, 100, 150, and 200 ms. **(C)** The paired-pulse ratio (PPR) was measured as the amplitude of the second EPSP divided by the first EPSP and is plotted against the interval between stimuli. *p < 0.05 and **p < 0.01 [Kolmogorov–Smirnov (K-S) test; *n* = 15 per genotype].

Next, we investigated in further detail the release probability in HD cortical neurons of zQ175 knock-in mice by recording eEPSCs in a postsynaptic neuron (Figure 3A); eEPSCs were evoked using a monopolar silver wire electrode, and the currents were then used to calculate the PPR. Figure 3B shows example recordings of a WT and HD neuron with increasing paired-pulse intervals. Given that the amplitude of an EPSC is largely dependent upon the number of synaptic vesicles released upon presynaptic stimulation, the ratio between the second EPSC amplitude and the first EPSC is widely used as a measure of release probability (Dobrunz and Stevens, 1997). Specifically, neurons with low release probability tend to have a larger available pool for the second stimulation, giving rise to paired-pulse facilitation (Debanne et al., 1996; Murthy et al., 1997). Therefore, the measured PPR is inversely correlated to release probability (Dobrunz and Stevens, 1997; Murthy et al., 1997).

Using the four pulse intervals shown in Figure 3B (i.e., 50, 100, 150, and 200 ms), we calculated the PPR for WT and HD neurons (Figure 3C). Our analysis (*n* = 15 per genotype) revealed that HD synapses have significantly smaller PPR values with a 100-ms (*p* = 0.005 from K-S test) and 150-ms (*p* = 0.017 from K-S test) interval between the first and second pulses compared with WT neurons. Moreover, the PPR in HD synapses was smaller than 1.0 at all interval tests, consistent with paired-pulse depression. Taken together, these data suggest that presynaptic terminals in HD cortical neurons of heterozygous zQ175 mice have increased release probability, consistent with our FM 1–43 results.

### Presynaptic Ca^2+^ Influx Is Increased in HD Cortical Neurons

Ca^2+^ influx into the presynaptic terminal triggers the release of neurotransmitter *via* the Ca^2+^-dependent fusion of synaptic vesicles (Luebke et al., 1993; Südhof, 2012). Moreover, the cumulative increase in presynaptic Ca^2+^ influx during high-frequency stimulation enhances the recruitment of synaptic vesicles for fusion (Wang and Kaczmarek, 1998; Hosoi et al., 2007). Thus, Ca^2+^ influx at presynaptic terminals is a key factor in determining the strength of neurotransmission (Murthy et al., 1997; Ermolyuk et al., 2012; Körber and Kuner, 2016).

**Figure 4:**
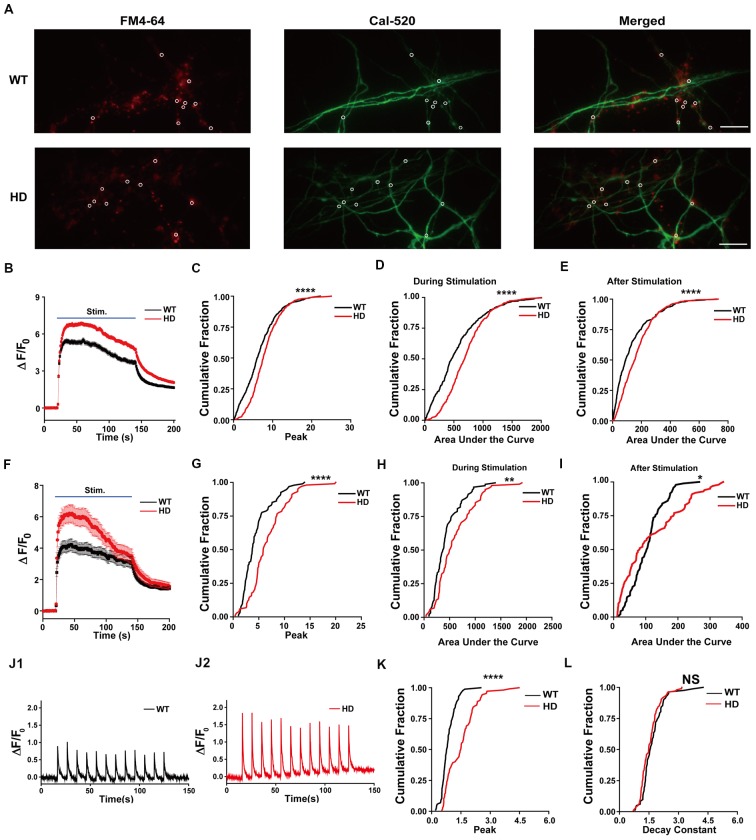
HD cortical neurons increase presynaptic Ca^2+^ influx. **(A)** Representative images of WT and HD cortical neurons loaded with FM 4–64 (to label presynaptic terminals) and Cal-520 (an ultrasensitive fluorescence-based Ca^2+^ indicator). Cal-520 fluorescence was measured at FM 4–64-positive puncta (white circles) as the change in fluorescence relative to baseline fluorescence (Δ*F/F*_0_). The scale bar represents 10 μm. **(B)** Average Δ*F/F*_0_ traces measured before, during, and after field stimulation with 1,200 1-ms pulses delivered at 10 Hz for 120 s; *n* = 474 boutons in WT (*N* = 16 experiments) and *n* = 571 boutons in HD neurons (*N* = 15 experiments). **(C)** The peak Δ*F/F*_0_ of Cal-520 during the electrical stimulation. **(D)** Area under the curve of fluorescence change (Δ*F/F*_0_) during the stimulation. **(E)** Area under the curve of fluorescence change (Δ*F/F*_0_) after stimulation. **(F)** Average Δ*F/F*_0_ traces measured before, during, and after field stimulation with 1,200 1-ms pulses delivered at 10 Hz for 120 s in the presence of 50 μM AP5, 10 μM NBQX, and 1 μM calciseptine; *n* = 94 boutons in WT (*N* = 6 experiments) and *n* = 102 boutons in HD neurons (*N* = 9 experiments). **(G)** The peak Δ*F/F*_0_ of Cal-520 in the presence of 50 μM AP5, 10 μM NBQX, and 1 μM calciseptine during the electrical stimulation. **(H)** Area under the curve of fluorescence change (Δ*F/F*_0_) in the presence of 50 μM AP5, 10 μM NBQX, and 1 μM calciseptine during the stimulation. **(I)** Area under the curve of fluorescence change (Δ*F/F*_0_) in the presence of 50 μM AP5, 10 μM NBQX, and 1 μM calciseptine after stimulation. **(J)** Representative traces of Δ*F/F*_0_ traces measured at 10 Hz imaging frequency before, during, and after stimulation with 12 1-ms pulses delivered at 0.1 Hz for 120 s in WT (*n* = 86 boutons and *N* = 7 experiments; **J1**) and HD (*n* = 107 boutons and *N* = 6 experiments) neurons **(J2)**. **(K)** Peak of Δ*F/F*_0_ measured after a single stimulus, averaged for single boutons. **(L)** Decay constant of Δ*F/F*_0_ after a single stimulus averaged for single boutons in WT (*n* = 86 boutons and *N* = 7 experiments) and HD (*n* = 107 boutons and *N* = 6 experiments) neurons. *p < 0.05, **p < 0.01, ****p < 0.0001 and NS, not significant (K-S test).

To investigate whether the increase in neurotransmission in HD neurons is mediated by an increase in presynaptic Ca^2+^ influx, we measured presynaptic Ca^2+^ transient during electrical field stimulation of neurons loaded with Cal-520-AM, an ultrasensitive fluorescence-based Ca^2+^ indicator well-suited for measuring Ca^2+^ level in subcellular compartments (Tada et al., 2014; Lock et al., 2015). Representative images of cultured WT and HD neurons co-loaded with FM 4–64 (to label presynaptic terminals) and Cal-520 are shown in Figure 4A. The fluorescence signal of Cal-520 was used to measure Ca^2+^ transients specifically at presynaptic terminals. Presynaptic Ca^2+^ was measured as the fluorescence change relative to resting fluorescence (Δ*F/F*_0_), which normalizes various factors, including differences in dye loading, in order to minimize possible artifacts associated with measuring raw fluorescence intensity (Chen et al., 2013; Lock et al., 2015). Average traces of Δ*F/F*_0_ in Cal-520-loaded WT and HD cortical neurons are shown in Figure 4B. Applying 1,200 field stimuli caused a significantly larger peak intensity in Δ*F/F*_0_ in presynaptic terminals of HD neurons compared to WT neurons [8.1 ± 0.17 (*n* = 571 boutons, *N* = 17 experiments for HD) vs. 6.7 ± 0.21 (*n* = 474 boutons, *N* = 16 experiments for WT), *p* = 1.51E-7, K-S test; Figure 4C], which indicates a significantly larger increase of Ca^2+^ influx in the presynaptic terminals of HD neurons during electrical field stimulation.

Next, we measured the entire increase in presynaptic Ca^2+^ during field stimulation by calculating the area under the curve for Δ*F/F*_0_ (Atucha et al., 2003; Reznichenko et al., 2012). The area under the curve for Δ*F/F*_0_ in the presynaptic terminals of HD neurons was significantly larger than that in WT terminals (714 ± 14.8 for HD vs. 559 ± 19.3 for WT, *p* = 1.37E-10; Figure 4D), implying that significantly more Ca^2+^ entered the presynaptic terminals in HD neurons compared with WT neurons during field stimulation. Moreover, presynaptic terminals of HD neurons had significantly more Ca^2+^ following field stimulation (170 ± 5.2 for HD vs. 126 ± 6.4 for WT, *p* = 1.10E-7; Figure 4E). These results suggest that HD cortical neurons of heterozygous zQ175 mice have increased Ca^2+^ influx, which may suggest a possible mechanism underlying the increased excitatory activity in HD. To confirm that the measured Δ*F/F*_0_ represents Ca^2+^ influx in presynaptic terminals, we repeated these experiments in the presence of 50 μM AP5 (an NMDA receptor antagonist), 10 μM NBQX (an AMPA receptor antagonist), and 1 μM calciseptine (an L-type voltage-gated Ca^2+^ channels blocker). The peak response, the area under the curve measured during 1,200 stimuli, and the area under the curve measured after the 1,200 stimuli were unaffected by the presence of these blockers (Figures 4F–I). Moreover, all of these parameters differed significantly WT an HD neurons (Figures 4F–I). These results confirm that we indeed measured Ca^2+^ in presynaptic terminals.

In order to examine the detailed mechanism underlying the increased Ca^2+^ influx in HD neurons, we also measured Ca^2+^ influx with high temporal resolution imaging. Figure 4J shows exemplar traces of Δ*F/F*_0_ measured using 10 Hz imaging while stimulating at 0.1 Hz in WT (Figure 4J1) and HD (Figure 4J2) neurons. The traces of Δ*F/F*_0_ show that the signal returned to baseline within 10 s of stimulation. We also measured the peak intensity of Δ*F/F*_0_ and the decay constant in response to a single stimulus. We found that the average peak Δ*F/F*_0_ after a single stimulus was significantly higher in HD neurons than in WT neurons [1.50 ± 0.075 for HD (*n* = 107 boutons, *N* = 6 experiments) vs. 0.85 ± 0.040 for WT (*n* = 86 boutons, *N* = 7 experiments), respectively, *p* < 1.0E-17, K-S test; Figure 4K], indicating that more Ca^2+^ ions enter the presynaptic terminals of HD neurons compared to WT neurons. The addition of more Ca^2+^ influx after each stimulus in the HD neurons led to the significantly larger influx of Ca^2+^ during 1,200 external stimuli shown in Figure 4C. Next, to investigate whether the clearance of Ca^2+^ is altered in HD neurons, we calculated the decay constant of Δ*F/F*_0_ by fitting the decay phase of Δ*F/F*_0_ to an exponential function. We found that the decay constant was similar between HD and WT neurons [1.54 ± 0.049 s for HD (*n* = 102 boutons) vs. 1.68 ± 0.064 s for WT (*n* = 82 boutons), *p* = 0.11, K-S test; Figure 4L], suggesting that Ca^2+^ clearance is not altered in HD neurons. The increased peak intensity in HD neurons, together with no change in Ca^2+^ clearance, suggests a possible association between the mutant HTT protein and voltage-gated Ca^2+^ channels during electrical stimulation.

### Loading HD Neurons With BAPTA-AM (a Ca^2+^ Chelator) Prevents Increased Synaptic Vesicle Release

**Figure 5:**
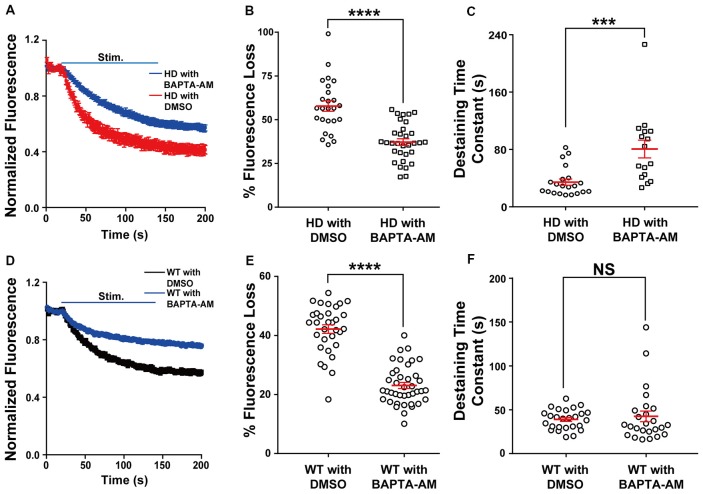
Loading HD and WT neurons with BAPTA-AM reduces the increased release of synaptic vesicles. **(A)** Average traces of normalized FM 1–43 fluorescence intensity in HD neurons loaded with BAPTA-AM (*n* = 32 boutons, *N* = 6 experiments) or vehicle dimethyl sulfoxide (DMSO; *n* = 25 boutons, *N* = 5 experiments). Where indicated, the neurons were stimulated with 1,200 1-ms field stimuli delivered at 10 Hz for 120 s. **(B)** The percent fluorescence loss of FM 1–43 in control-treated and BAPTA-AM-loaded HD cortical neurons. **(C)** The time constant of FM 1–43 destaining in control-treated and BAPTA-AM-loaded HD cortical neurons. **(D)** Average traces of normalized FM 1–43 fluorescence intensity in WT neurons loaded with BAPTA-AM (*n* = 40 boutons, *N* = 5 experiments) or vehicle (DMSO; *n* = 32 boutons, *N* = 5 experiments). Where indicated, the neurons were stimulated with 1,200 1-ms field stimuli delivered at 10 Hz for 120 s. **(E)** The percent fluorescence loss of FM 1–43 in control-treated and BAPTA-AM-loaded WT cortical neurons. **(F)** The time constant of FM 1–43 destaining in control-treated and BAPTA-AM-loaded WT cortical neurons. NS, not significant, ***p < 0.001, and ****p < 0.0001 (independent two-tailed Student’s *t*-test).

Next, we directly examined the role of presynaptic Ca^2+^ on neurotransmission in HD cortical neurons by loading neurons with the membrane-permeable Ca^2+^ chelator BAPTA-AM (Tsien, 1981) and measuring vesicle fusion in single FM 1–43-loaded presynaptic terminals. We used 200 nM BAPTA for these experiments, as this chelator has been reported to protect neurons against excitotoxicity (Tymianski et al., 1993, 1994; Abdel-Hamid and Tymianski, 1997). As shown in Figure 5A, HD cortical neurons loaded with BAPTA-AM had significantly reduced the release of vesicles compared to control HD neurons (loaded with DMSO); this reduction was reflected in both the significant average fluorescence loss [37.9 ± 2.0% (*n* = 32 boutons, *N* = 6 experiments with BAPTA-AM) vs. 57.8 ± 3.0% (*n* = 25 boutons, *N* = 5 experiments with DMSO), *p* = 1.33E-7, independent two-tailed Student’s *t*-test; Figure 5B] and the increased time constant of FM 1–43 destaining [80.6 ± 12.4 s (*n* = 16 boutons) with BAPTA-AM vs. 34.4 ± 4.4 s (*n* = 21 boutons) with DMSO, *p* = 0.000428; Figure 5C]. Interestingly, loading HD neurons with BAPTA reduced fluorescence loss to WT levels [37.9 ± 2.0% vs. 41.7 ± 1.4% (WT), respectively]. The application of BAPTA-AM to WT neurons also decreased the fluorescence loss [23.1 ± 1.0% (*n* = 40 boutons, *N* = 5 experiments with BAPTA-AM) vs. 42.2 ± 1.5% (*n* = 32 boutons, *N* = 5 experiments with DMSO), *p* = 8.29E-17; Figures 5D,E], but did not decrease the destaining time constant [42.4 ± 6.2 s (*n* = 25 boutons with BAPTA-AM) vs. 39.9 ± 2.2% (*n* = 26 boutons with DMSO), *p* = 0.59, independent two-tailed Student’s *t*-test; Figure 5F]. These results indicate that restricting Ca^2+^ influx at presynaptic terminals prevents the increased neurotransmission in HD cortical neurons of heterozygous zQ175 mice.

### Blocking N-type Voltage-Gated Ca^2+^ Channels Prevents Increased Synaptic Vesicle Release and Presynaptic Ca^2+^ Influx in HD Neurons

**Figure 6:**
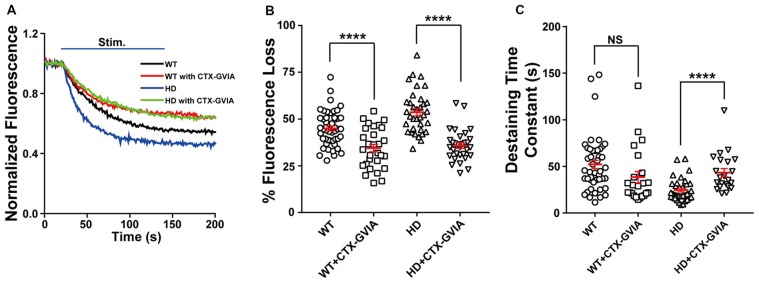
Blocking N-type voltage-gated Ca^2+^ channels reduces the increased release of synaptic vesicles in HD cortical neurons. **(A)** Average traces of normalized FM 1–43 fluorescence intensity in untreated HD (*n* = 36 boutons, *N* = 4 experiments) and WT cortical neurons (*n* = 49 boutons, *N* = 5 experiments), HD (*n* = 31 boutons, *N* = 5 experiments) and WT cortical neurons (*n* = 29 boutons, *N* = 4 experiments) treated with 100 nM ω-conotoxin GVIA (Ctx-GVIA). Where indicated, 1,200 1-ms field stimuli were delivered at 10 Hz for 120 s.** (B)** The percent fluorescence loss of FM 1–43 in untreated and ω-conotoxin GVIA-treated HD and WT neurons. The average change in fluorescence loss in response to ω-conotoxin GVIA treatment was considerably smaller in WT cortical neurons (10.5%) compared with HD neurons (17.8%). **(C)** The time constant of FM 1–43 destaining in untreated and ω-conotoxin GVIA-treated HD and WT cortical neurons. The destaining rates of FM 1–43 in WT cortical neurons with ω-conotoxin GVIA were not significantly different from those without ω-conotoxin GVIA (*p* > 0.08). NS, not significant, and ****p < 0.0001 (independent two-tailed Student’s *t*-test).

Evoked vesicle fusion requires presynaptic Ca^2+^ influx through voltage-gated Ca^2+^ channels, which are coupled to the fusion machinery at presynaptic terminals (Vázquez and Sánchez-Prieto, 1997; Simms and Zamponi, 2014). In the central nervous system, presynaptic Ca^2+^ influx is mediated primarily by N-type (Ca_v_2.2) and P/Q-type (Ca_v_2.1) voltage-gated Ca^2+^ channels (Zamponi, 2003; Catterall, 2011). However, several reports have suggested that N-type voltage-gated Ca^2+^ channels can be affected by mutant HTT protein. That is, mutant HTT proteins were reported to modulate N-type voltage-gated Ca^2+^ channels by their interaction with N-type voltage-gated Ca^2+^ channels and binding proteins (Miller et al., 2003; Swayne et al., 2005; Silva et al., 2017), or the expression level of N-type voltage-gated Ca^2+^ channels in the plasma membrane (Silva et al., 2017). To test the role of N-type voltage-gated Ca^2+^ channels in the increased Ca^2+^ influx and neurotransmission measured in HD cortical neurons, we treated HD neurons with 100 nM ω-conotoxin GVIA (a highly selective blocker of N-type voltage-gated Ca^2+^ channels). We found that ω-conotoxin GVIA decreased the release of FM 1–43 during field stimulation (Figure 6A), reflected by a significant decrease in fluorescence loss [53.6 ± 1.9% (*n* = 36 boutons, *N* = 4 experiments without ω-conotoxin GVIA) vs. 34.8 ± 1.5% (*n* = 31 boutons, *N* = 5 experiments with ω-conotoxin GVIA), *p* = 7.18E-10, independent two-tailed Student’s *t*-test; Figure 6B]. Treatment with ω-conotoxin GVIA also significantly increased the time constant of FM 1–43 destaining [24.5 ± 2.0 s (*n* = 35 boutons without ω-conotoxin GVIA) vs. 43.3 ± 4.4 s (*n* = 22 boutons with ω-conotoxin GVIA), *p* = 0.000063; Figure 6C].

We also treated WT neurons with ω-conotoxin GVIA and measured the release of FM 1–43 during field stimulation (Figure 6A). We found a significant decrease in fluorescence loss of FM 1–43 with ω-conotoxin GVIA [45.0 ± 1.3% (*n* = 49 boutons, *N* = 5 experiments without ω-conotoxin GVIA) vs. 34.5 ± 2.0% (*n* = 29 boutons, *N* = 4 experiments with ω-conotoxin GVIA), *p* = 0.000019; Figures 6A,B]. However, the effect of application of ω-conotoxin GVIA on fluorescence loss of HD neurons was significantly different compared with WT neurons (*p* = 0.0339, two-way ANOVA analysis). In addition, application of ω-conotoxin GVIA in WT cortical neurons did not significantly affect the time constant of FM 1–43 destaining in WT cortical neurons [52.1 ± 4.5 s (*n* = 45 boutons without ω-conotoxin GVIA) vs. 38.4 ± 6.2 s (*n* = 23 boutons with ω-conotoxin GVIA), *p* = 0.0814; Figure 6C], suggesting that blocking N-type voltage-gated Ca^2+^ channels does not affect vesicle release kinetics in WT cortical neurons. The effect of application of ω-conotoxin GVIA on vesicle release kinetics of HD neurons was significantly different compared with WT neurons (*p* = 0.0006, two-way ANOVA analysis).

**Figure 7:**
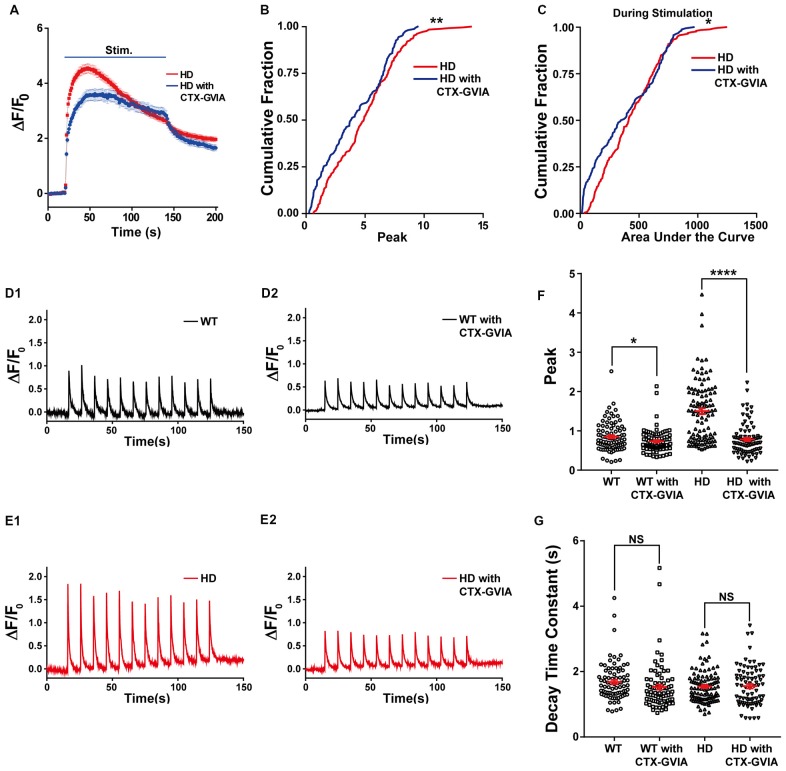
Blocking N-type voltage-gated Ca^2+^ channels reduces Ca^2+^ influx in HD cortical neurons. **(A)** Average Δ*F/F*_0_ traces of Cal-520 measured in the untreated (*n* = 254 boutons, *N* = 6 experiments) and ω-conotoxin GVIA-treated HD neurons (*n* = 162 boutons, *N* = 6 experiment). Where indicated, 1,200 1-ms field stimuli were delivered at 10 Hz for 120 s.** (B)** The peak Δ*F/F*_0_ of Cal-520 during electrical stimulation in the untreated and ω-conotoxin GVIA-treated HD neurons. **p < 0.01 (K-S test).** (C)** The area under the curve of Cal-520 during electrical stimulation in the untreated and ω-conotoxin GVIA-treated HD neurons. *p < 0.05 (K-S test). **(D)** Representative traces of Δ*F/F*_0_ traces measured at 10 Hz imaging frequency before, during, and after stimulation with 12 1-ms pulses delivered at 0.1 Hz for 120 s in WT neurons in the absence **(D1)** or presence of ω-conotoxin GVIA **(D2)**. **(E)** Representative traces of Δ*F/F*_0_ traces measured at 10 Hz imaging frequency before, during, and after stimulation with 12 1-ms pulses delivered at 0.1 Hz for 120 s in HD neurons in the absence **(E1)** or presence of ω-conotoxin GVIA **(E2)**. **(F)** Peak of Δ*F/F*_0_ measured after a single stimulus, averaged for single boutons of WT and HD neurons in the presence or absence of ω-conotoxin GVIA (CTX-GVIA). *p < 0.05 and ****p < 0.0001 (independent two-tailed Student’s *t*-test). **(G)** Decay constant of Δ*F/F*_0_ after a single stimulus averaged for single boutons in WT and HD neurons in the presence or absence of ω-conotoxin GVIA (CTX-GVIA). NS, not significant (independent two-tailed Student’s *t*-test).

In addition, treating HD neurons with ω-conotoxin GVIA reduced the increase in presynaptic Ca^2+^ influx (Figure 7A); treating HD neurons with ω-conotoxin GVIA significantly reduced both the peak increase in Δ*F/F*_0_ [4.9 ± 0.17 (*n* = 254 boutons, *N* = 6 experiments without ω-conotoxin GVIA) vs. 4.1 ± 0.21 (*n* = 162 boutons, *N* = 6 experiments with ω-conotoxin GVIA), *p* = 0.00232, K-S test; Figure 7B] and the area under the curve during the field stimulation [435 ± 15.6 without ω-conotoxin GVIA vs. 383 ± 21.6 with ω-conotoxin GVIA, *p* = 0.047; Figure 7C].

Next, we tested whether the decreased Ca^2+^ influx during 1,200 external stimuli in HD neurons in the presence of ω-conotoxin GVIA is caused by decreased Ca^2+^ influx by a single stimulus, we used high temporal resolution imaging to measure the average peak Δ*F/F*_0_ in response to a single stimulus. The application of ω-conotoxin GVIA to HD neurons decreased the average peak Δ*F/F*_0_ significantly [1.50 ± 0.075 for HD without ω-conotoxin GVIA (Figure 7E1; *n* = 107 boutons, *N* = 6 experiments) vs. 0.80 ± 0.042 for HD with ω-conotoxin GVIA (Figure 7E2; *n* = 86 boutons, *N* = 5 experiments), *p* = 2.1E-13, independent two-tailed Student’s *t*-test; Figures 7E,F]. The application of ω-conotoxin GVIA to WT neurons also decreased the average peak Δ*F/F*_0_ significantly [0.85 ± 0.040 for WT without ω-conotoxin GVIA (Figure 7D1; *n* = 86 boutons, *N* = 7 experiments) vs. 0.73 ± 0.032 for WT with ω-conotoxin GVIA (Figure 7D2; *n* = 84 boutons, *N* = 5 experiments), *p* = 0.028, independent two-tailed Student’s *t*-test; Figures 7D,F], but the effect was significantly smaller in WT neurons than in HD neurons (*p* = 4.2E-8, two-way ANOVA). We also calculated the decay constant of Δ*F/F*_0_ by fitting the of Δ*F/F*_0_ traces to an exponential function. The decay constants in HD and WT neurons were unaffected by the presence of ω-conotoxin GVIA [1.54 ± 0.049 s without ω-conotoxin GVIA (*n* = 102 boutons, *N* = 6 experiments) vs. 1.56 ± 0.066 s with ω-conotoxin GVIA (*n* = 81 boutons, *N* = 5 experiments) for HD, *p* = 0.89; 1.68 ± 0.064 s without ω-conotoxin GVIA (*n* = 82 boutons, *N* = 7 experiments) vs. 1.53 ± 0.077 s with ω-conotoxin GVIA (*n* = 82 boutons, *N* = 5 experiments) for WT, *p* = 0.12, independent two-tailed Student’s *t*-test; Figure 7G], suggesting that the clearance of Ca^2+^ in neurons is not affected by the presence of ω-conotoxin GVIA in either HD or WT neurons. These results indicate that N-type voltage-gated Ca^2+^ channels likely play an important role in the increased Ca^2+^ influx in HD cortical neurons of heterozygous zQ175.

**Figure 8:**
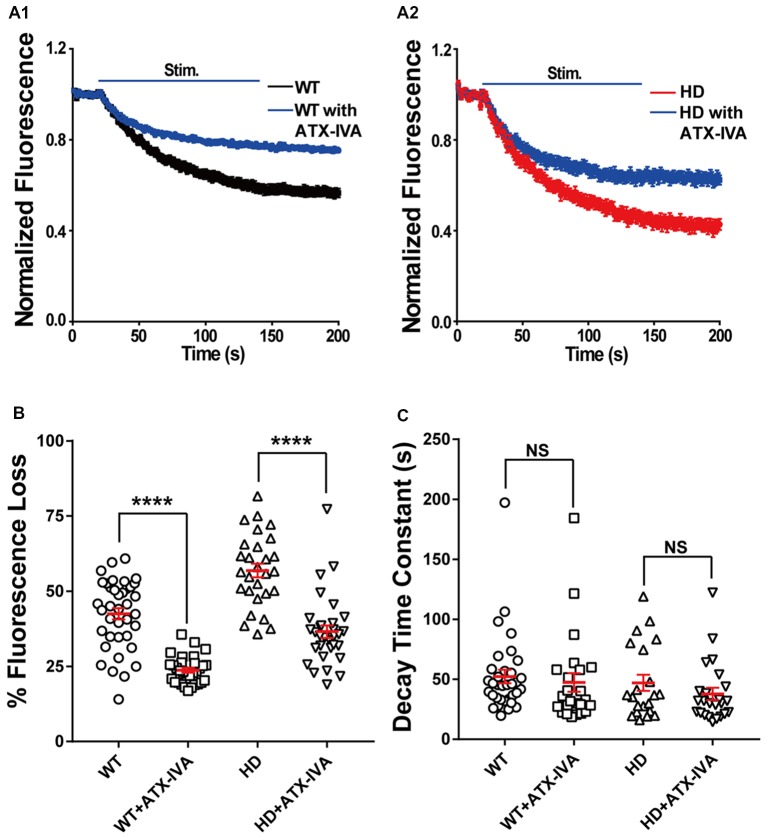
Blocking P/Q-type voltage-gated Ca^2+^ channels does not affect the increased release of synaptic vesicles in HD cortical neurons compared to WT neurons. **(A)** Average traces of normalized FM 1–43 fluorescence intensity in untreated (*n* = 36 boutons, *N* = 4 experiments) and 200 nM ω-agatoxin IVA(ATX-IVA)-treated WT neurons (*n* = 31 boutons, *N* = 5 experiments; **A1**), and untreated (*n* = 36 boutons, *N* = 4 experiments) and 200 nM ω-agatoxin IVA(ATX-IVA)-treated HD neurons (*n* = 31 boutons, *N* = 5 experiments; **A2**). Where indicated, a train of 1,200 1-ms field stimuli was applied at 10 Hz for 120 s. **(B)** The percent loss of FM 1–43 fluorescence measured in WT and HD neurons in the presence or absence of ATX-IVA. Note that ω-agatoxin IVA caused a similar change in percent fluorescence loss in WT and HD neurons (18.7% and 20.3%, respectively). **(C)** The time constant of FM1–43 destaining measured in WT and HD neurons in the presence or absence of ATX-IVA. ****p < 0.0001 and NS, not significant (independent two-tailed Student’s *t*-test).

Given that P/Q-type voltage-gated Ca^2+^ channels are also a major source of Ca^2+^ influx at presynaptic terminals, driving synaptic transmission (Wheeler et al., 1994), we also examined the role of P/Q-type voltage-gated Ca^2+^ channels in the increased synaptic vesicle release in HD cortical neurons by measuring FM 1–43 destaining in the absence and presence of 200 nM ω-agatoxin IVA (a selective blocker of P/Q-type voltage-gated Ca^2+^ channels). Application of ω-agatoxin IVA decreased fluorescence loss in WT (Figure 8A1) and HD (Figure 8A2) cortical neurons significantly compared with untreated ones (Figures 8A,B). However, the decrease of FM 1–43 in the presence of ω-agatoxin IVA was similar regardless of genotype [18.8% (WT) vs. 20.3% (HD), Figure 8B] and the effect of ω-agatoxin on fluorescence loss of HD neurons was not significantly different compared with WT neurons (*p =* 0.669, two-way ANOVA analysis). Moreover, ω-agatoxin IVA did not affect the time constant of FM 1–43 destaining in both WT and HD neurons significantly (Figure 8C) and the effect of ω-agatoxin IVA on vesicle release kinetics of HD neurons was not significantly different compared WT neurons (*p* = 0.741, two-way ANOVA analysis). Taken together, these results suggest that N-type voltage-gated Ca^2+^ channels—and not P/Q-type voltage-gated Ca^2+^ channels—play an important role in the increased release of synaptic vesicles at presynaptic terminals of HD cortical neurons in heterozygous zQ175 mice.

## Discussion

It has been suggested that synaptic dysfunction (i.e., “synaptopathy”) is involved in many neurodegenerative diseases, including Alzheimer’s disease and HD (Li et al., 2003; Bae and Kim, 2017; Tyebji and Hannan, 2017). Recent work in the field of HD suggests that altered synaptic transmission may play an important role in the pathogenesis of HD (Li et al., 2003; Sepers and Raymond, 2014; Raymond, 2017; Tyebji and Hannan, 2017). The altered synaptic function in corticostriatal synapses is a prodromal symptom and can trigger the death of vulnerable striatal neurons in HD. The mechanism that underlies altered synaptic transmission at presynaptic terminals in HD neurons remains elusive. In particular, the release of synaptic vesicles at single presynaptic terminals is poorly understood. Here, we performed real-time measurements of synaptic vesicles release at single presynaptic terminals in primary cultured HD cortical neurons obtained from a knock-in mouse model of HD (zQ175). We found that compared to WT neurons, HD cortical neurons of heterozygous zQ175 mice have increased release of synaptic vesicles and presynaptic Ca^2+^ influx. We also found that the increased neurotransmission and presynaptic Ca^2+^ influx were significantly reduced by loading the neurons with BAPTA-AM (a Ca^2+^ chelator) or treating neurons with ω-conotoxin GVIA (a highly selective N-type voltage-gated Ca^2+^ channel blocker).

Our finding of increased neurotransmission in primary cultured cortical neurons of a knock-in model of HD (zQ175) is consistent with previous reports of increased neurotransmission in acute cortical slices from presymptomatic YAC128 mice (Joshi et al., 2009) and at the neuromuscular junction in R6/1 mice (Rozas et al., 2011), two mouse models of HD. Our results are also consistent with increased neurotransmitter release in a *Drosophila* model of HD (Romero et al., 2008) and the recent report of increased glutamate release in striatal synaptosomes isolated from 6-month-old 140Q knock-in mice (Valencia et al., 2013).

Intracellular Ca^2+^ regulates a wide range of signaling pathways (Brini et al., 2014). Altered Ca^2+^ regulation in neurons has been reported in many neurodegenerative diseases, including Alzheimer’s disease, Parkinson’s disease, and HD (Bezprozvanny, 2009). With respect to HD, increased somatic Ca^2+^ transients were reported in striatal neurons in several HD mouse models (Tang et al., 2005; Fernandes et al., 2007; Rosenstock et al., 2010). Here, we report that Ca^2+^ influx at single presynaptic terminals is increased in HD cortical neurons of heterozygous zQ175 mice, and this increased Ca^2+^ influx is associated with increased neurotransmission. Together with other reports suggesting that mutant HTT proteins modulate N-type voltage-gated Ca^2+^ channels through their interaction with N-type voltage-gated Ca^2+^ channels and their binding proteins (Miller et al., 2003; Swayne et al., 2005; Silva et al., 2017), our results suggest that N-type voltage-gated Ca^2+^ channels, play an important role in the increased presynaptic Ca^2+^ influx and synaptic vesicle release in cortical neurons from HD mice. However, it is possible that other Ca^2+^ channels may contribute to the increased Ca^2+^ influx in HD neurons (Silva et al., 2017). Several other mechanisms, including InsP_3_R1 and store-operated Ca^2+^ entry (SOCE), may increase the level of Ca^2+^ inside HD neurons (Miller and Bezprozvanny, 2010; Raymond, 2017). Therefore, additional experiments are needed to better understand the mechanism underlying increased presynaptic Ca^2+^ influx in cortical neurons in HD. In particular, investigating the effects of increased presynaptic Ca^2+^ influx, including one from N-type voltage-gated Ca^2+^ channels, on the neurodegeneration of HD neurons will contribute to identifying potential therapeutic targets for HD.

Although our data suggest that increased presynaptic Ca^2+^ influx contributes to the increased neurotransmission in zQ175 mice, we cannot exclude the possibility that other mechanisms might play a role, including the binding of mutant HTT protein to regulatory proteins that control vesicle fusion. For example, the mutant HTT protein can bind vesicular proteins (DiFiglia et al., 1995; Liévens et al., 2002), thereby increasing neurotransmitter release (Valencia et al., 2013).

Based on our findings, we propose a model in which increased excitatory neurotransmission in cortical neurons due to increased presynaptic Ca^2+^ influx *via* the interaction of N-type voltage Ca^2+^ channels with mutant HTT proteins can increase the activation of NMDARs, thereby inducing excitotoxicity in cortical and striatal neurons, ultimately leading to the progressive loss of these neurons in HD. Importantly, this model may explain the vulnerability of MSNs in the indirect pathway; these MSNs would be particularly susceptible to increased excitatory neurotransmission, as they are more depolarized than MSNs in the direct pathway from the cortex (Gertler et al., 2008) and are therefore more excitable. Thus, our model may explain the increased firing (Kreitzer and Malenka, 2007; Cepeda et al., 2008) and increased vulnerability (Cepeda et al., 2008) of MSNs in the indirect pathway in HD. Our model also suggests that the increase in excitatory neurotransmission causes the hyperactivity of MSNs that proceeds neural degeneration and death (Raymond et al., 2011). In addition, our model provides a possible explanation for the recent report about the contribution of presynaptic terminals from cortical neurons to HD at reconstituted corticostriatal synapses (Virlogeux et al., 2018); the increased release of excitatory neurotransmitters from the presynaptic terminals of cortical neurons may lead to degeneration of striatal neurons. Future experiments of corticostriatal synapses and organotypic slices at different ages of mice will help to identify the detailed mechanisms about how increased excitatory neurotransmission contribute to neurodegeneration of MSNs as HD progresses.

In summary, we report the real-time measurements of synaptic vesicle release and Ca^2+^ influx at single presynaptic terminals during electrical field stimulation in primary cortical neurons obtained from a transgenic knock-in mouse model of HD (zQ175). We found that HD cortical neurons from heterozygous zQ175 mice had increased neurotransmission and presynaptic Ca^2+^ influx compared to WT neurons. These increases were reduced by treating the neurons with an intracellular Ca^2+^ chelator or by pharmacologically blocking N-type voltage-gated Ca^2+^ channels, thereby providing a possible new therapeutic target for HD. Our findings also suggest that the increase in neurotransmission and/or presynaptic Ca^2+^ influx may underlie the excitotoxicity in vulnerable neurons in HD and the eventual loss of these neurons.

## Author Contributions

SC, CY, and HP designed the experiments. SC, CY, LR and CL performed the experiments. SC, CY, and XQ provided the analysis programs. SC, CY, HR and HP wrote the manuscript.

## Conflict of Interest Statement

The authors declare that the research was conducted in the absence of any commercial or financial relationships that could be construed as a potential conflict of interest.
