# Supplementary material for: Altered Synaptic Vesicle Release and Ca2+ Influx at Single Presynaptic Terminals of Cortical Neurons in a Knock-in Mouse Model of Huntington’s Disease
Source: Front Mol Neurosci. 2018 Dec 24;11:478. doi: 10.3389/fnmol.2018.00478 (PMC6311661; doi:10.3389/fnmol.2018.00478)
Supplement: Supplementary file 1 [file Data_Sheet_1.PDF]

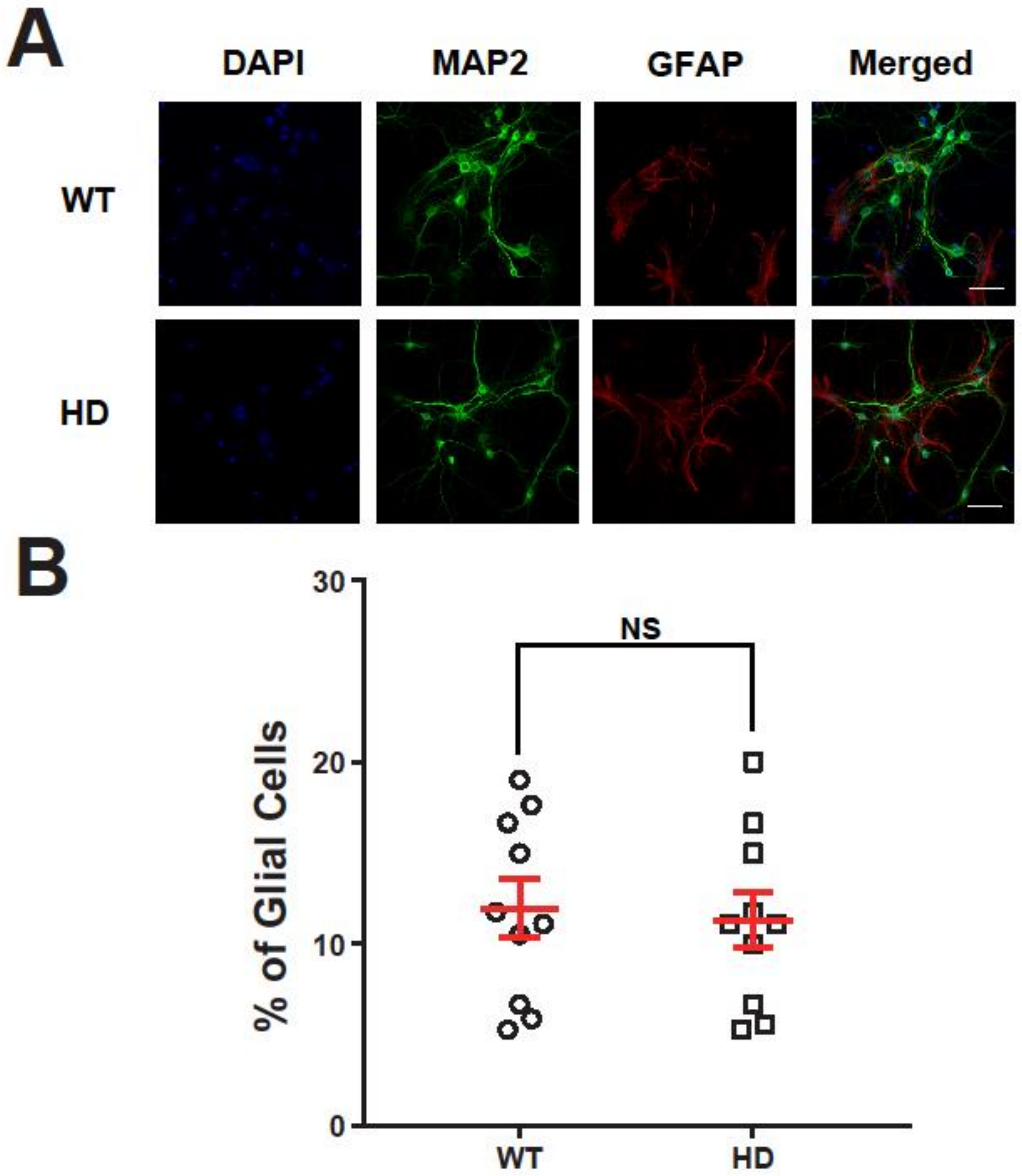

Supplemental Figure S1| Distribution of glial cells in primary neuronal cultures. **(A)** Representative confocal images of primary WT and HD cultures immunostained for the neuronal marker MAP2 (green) and the glial cell marker GFAP (red); the nuclei were counterstained with DAPI (blue). The scale bars represent 50 μm. **(B)**. The percentage of glial cells in WT and HD primary culture. The percentage between WT and HD showed no significant differences (NS: not significant, p=0.77, independent two-tailed Student's t-test).

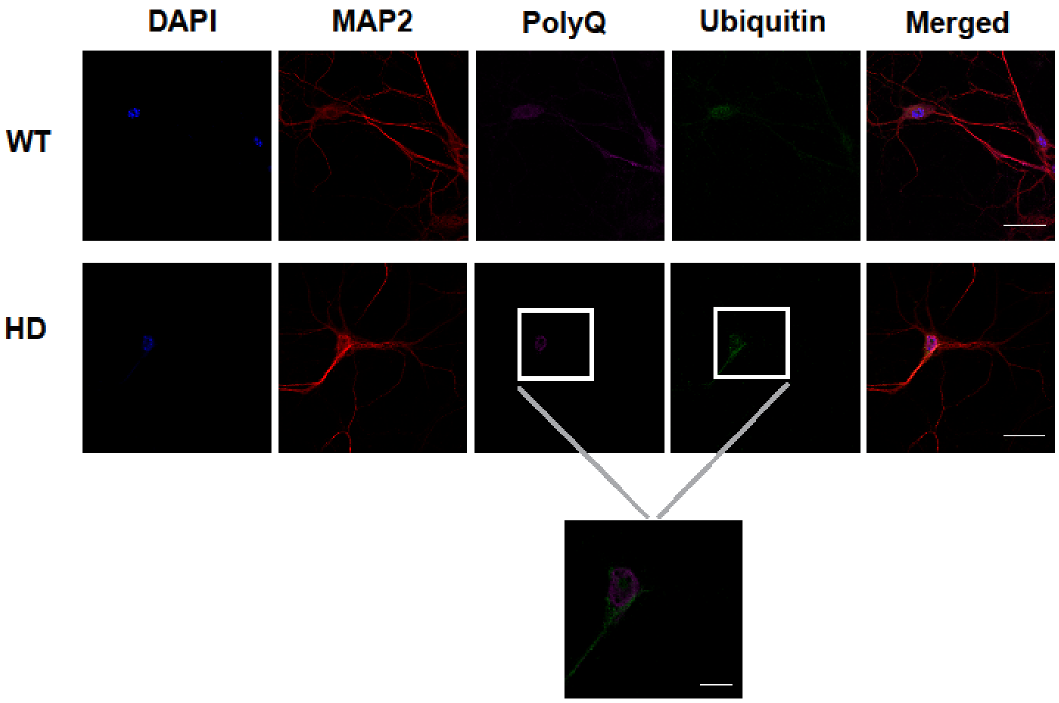

Supplemental Figure S2| Representative confocal images of cultured WT and HD cortical neurons immunostained for MAP2 (red), polyglutamine (polyQ, magenta), and ubiquitin (green); the nuclei were counterstained with DAPI (blue). The expanded view in the inset shows no co-localization between polyglutamine (magenta) and ubiquitin (green), confirming a lack of visible inclusion bodies. The scale bars s 30 μm; the scale bar in the inset represents 10 μm.

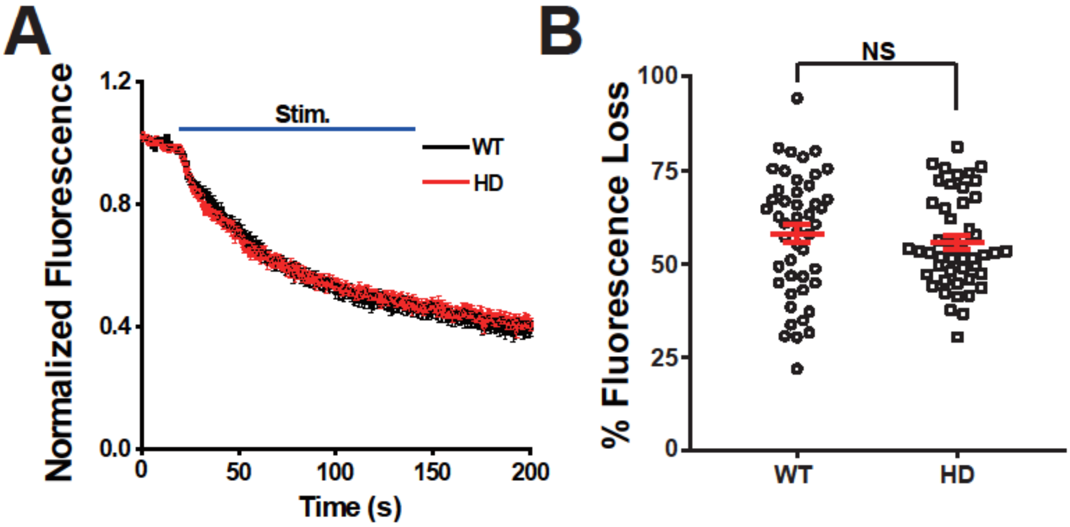

Supplemental Figure S3| The release of inhibitory synaptic vesicles does not differ significantly between WT and HD cortical neurons. **(A)** Average traces of normalized VGAT-CypHer5E fluorescence intensity measured in WT cortical neurons (n = 47 boutons, N = 5 experiments) and HD cortical neurons (n = 47 boutons, N = 6 experiments). Where indicated, a train of 1200 1-ms field stimuli was applied at 10 Hz for 120 s. **(B)** The percent fluorescence loss in VGAT-CypHer5E in WT and HD cortical neurons after 1200 stimuli. NS: not significant (p=0.47; independent two-tailed Student's t-test).
